# Effective Design of the Graded Strut of BCC Lattice Structure for Improving Mechanical Properties

**DOI:** 10.3390/ma12132192

**Published:** 2019-07-08

**Authors:** Long Bai, Changyan Yi, Xiaohong Chen, Yuanxi Sun, Junfang Zhang

**Affiliations:** State Key Laboratory of Mechanical Transmission, Chongqing University, Chongqing 400044, China

**Keywords:** mechanical properties, lattice structure, graded density, porous structure, sandwich structure

## Abstract

In order improve the poor mechanical properties of the body-centred cubic (BCC) lattice structure, which suffers from the stress concentration effects at the nodes of the BCC unit cell, a graded-strut design method is proposed to increase the radii corner of the BCC nodes, which can obtain a new graded-strut body-centred cubic (GBCC) unit cell. After the relative density equation and the force model of the structure are obtained, the quasi-static uniaxial compression experiments and finite element analysis (FEA) of GBCC samples and BCC samples are performed. The experimental results show that for the fabricated samples with the same relative density, the GBCC can increase the initial stiffness by at least 38.20%, increase the plastic failure strength by at least 34.12%, compared with the BCC. Coupled experimental and numerical results not only suggest that the GBCC has better mechanical and impact resistance properties than the BCC, but also indicate that as the radii corner increases, the stress concentration effect at the node and the mechanical properties will be improved, which validates the proposed design method for graded-strut unit cells and can provide guidance for the design and future research on ultra-light lattice structures in related fields.

## 1. Introduction

With the rapid development of additive manufacturing technology [1,2,3,4,5,6,7,8,9] and metal lattice structure materials [10], lightweight and high-strength multi-functional materials with periodic cellular unit cells that simulate the lattice configuration of a crystal molecule lattice have become the research trends of new materials in recent years. Amongst these, the body-centered cubic (BCC) lattice structure is widely used because of its simple configuration, isotropic structure and excellent adaptability to the selective laser melting (SLM) fabrication process [11].

The BCC lattice structure is a crystal molecular body structure with equal-cross-section homogeneous struts. Previous studies have focused mainly on its mechanical properties and failure mechanisms. For examples, Ushijima et al. [12], Gümrük et al. [13] and Feng et al. [14] established theoretical prediction models of the BCC unit cell in terms of the initial stiffness and the plastic collapse strength under various stress conditions. Ptochos et al. [15] proposed the shear modulus prediction model of the irregular BCC lattice structure (cuboid BCC unit cell) when the structure is shear-loaded in the *X*-, *Y*- and *Z*-directions. Bai et al. [16] solved the multi-objective optimization mathematical model of the unit cell size using the ideal point method, and obtained the optimal structure size of the body-centered tetragonal (BCT) unit cell. After establishing the theoretical prediction model for the mechanical properties of BCC, it is necessary to compare and analyze the actual mechanical performance. Shen et al. [17], Gümrük et al. [18] and Mckown et al. [19] examined the differences in mechanical properties of BCC, body-centred cubic with (vertical) *Z*-struts (BCCZ) and other lattice structures under various loading scenarios such as three-point bending, low-speed impact, compression, shear, tension and combined loading. Beharic et al. [20] compared the drop weight impact characteristics and their relationships between the quasi-static properties of BCC, the re-entrant auxetic and octet-truss structures at different temperatures. Mines et al. [21] studied the drop weight impact performance of the Ti6Al4V BCC lattice sandwich structure, SS316L BCC lattice sandwich structure and conventional aluminum honeycomb. Vincenzo et al. [22], Maskery et al. [23] and Mazur et al. [24] investigated the effects of the BCC unit cell size and quantity, unit cell relative density, aspect ratio and structural boundary condition on the compression performance by combining theoretical calculations, finite-element simulations and experiments, and compared the BCC structure with other typical structures such as face-centred cubic with (vertical) *Z*-struts (FCCZ) and BCCZ.

In order to further improve the mechanical properties of the BCC lattice structure in a specific application environment based on the mechanical performance, it is essential to study the failure mechanism of the structure. Ushijima et al. [12] and Arash et al. [25] discovered that the leading cause of the lattice structure failure is the plastic hinge near the nodes. The strut rotates around the plastic hinge and eventually endangers the plastic hinges into a local plastic shear band in the strut. Xiang et al. [26] and Arash et al. [27] noted that failure modes of samples are related to the vulnerable local area instead of the size of the unit cell, the overall structure and orientation of the sample regarding its building direction. Leary et al. [28], Gorny et al. [29] and Fei et al. [30] further revealed that the low compliance and the failure mode of the BCC lattice structures are related to the stress concentration at the nodes, process-induced pores and microstructure, which can be adjusted by the heat treatment. Pei [31] and Smith et al. [11] also noted that the plastic deformation of the lattice structure began near the node of the strut and that the local stress of the lattice structure was concentrated near the nodes.

The stress concentration at the nodes of the BCC uniform unit cell severely weakens the mechanical properties of the BCC lattice sandwich structure. To improve the mechanical properties of this structure, some scholars have proposed an overall graded-density design. Montazerian et al. [32], Yan et al. [33], Dalaq et al. [34], Liu et al. [35], Maskery et al. [36], Abueidda et al. [37] and Lei Yang et al. [38,39,40] adopted triply periodic minimal surfaces (TPMS) as the tool for designing internal pore architecture of porous lattice structure with smooth joints and curvatures. Daynes et al. [41] obtained the density distribution of the beam (composed of the lattice sandwich structure) with a three-point bending load and filled it with different-density BCC unit cells according to various density distributions, thereby obtaining a spatially graded-density beam with the influence of graded density on the structure stiffness and strength studied. Maskery et al. [42,43] designed a graded-density layer lattice structure by changing the relative density of the unit cell in each layer. They analyzed the effect of graded density on the structural compressive performance, energy absorption performance and failure mode, and found that the graded-density layer lattice structure can improve the mechanical properties of the cellular sandwich material, while the effect of the stress concentration at the nodes of the unit cell in a lattice structure is not addressed. Al-Saedi et al. [44] adopt a layer-by-layer functionally graded design method for F2BCC (the F2BCC lattice unit cell consists of 12 solid struts of circular cross-section, which they intersect at a 45° angle to vertical, four at the cell center, and eight at the four faces of the cell (two struts at each face)) which is made of Al-12Si aluminum alloy and manufactured with the SLM process. The functionally graded lattice structures were found to exhibit distinct mechanical properties and energy absorption capability when compared to the uniform lattice structure through experimental compression tests and finite element analysis (FEA). Based on the results obtained on the microCT-based load simulation, Plessis et al. [45] pointed out that adding material at areas of high stress can assist in producing stronger parts.

At present, there are few studies on improving the stress concentration effect at the unit cell node of lattice structure. Local stress concentration of the BCC lattice structure is induced by small radii corners of nodes [46], and researches on the failure mechanism of the BCC lattice structure show that the stress concentration is the main cause [31]. If the radii corner of the node increase, the stress concentration of the structure can be significantly reduced. Therefore, to relieve the stress concentration of the BCC unit cell node, a new type of lattice structure named the graded-strut body-centered cubic (GBCC) lattice structure is proposed in this paper by designing the graded cross section (graded-strut design) of the BCC cell strut.

The rest of this paper is organized as follows: Section 2 introduces the design of the GBCC unit cell. Section 3 establishes the GBCC theoretical model. Section 4 introduces the experimental and finite element simulation details. Section 5 discusses the results and Section 6 concludes the paper.

## 2. Design of the GBCC Unit Cell

### 2.1. Stress Analysis of the GBCC Unit Cell Node

**Figure 1:**
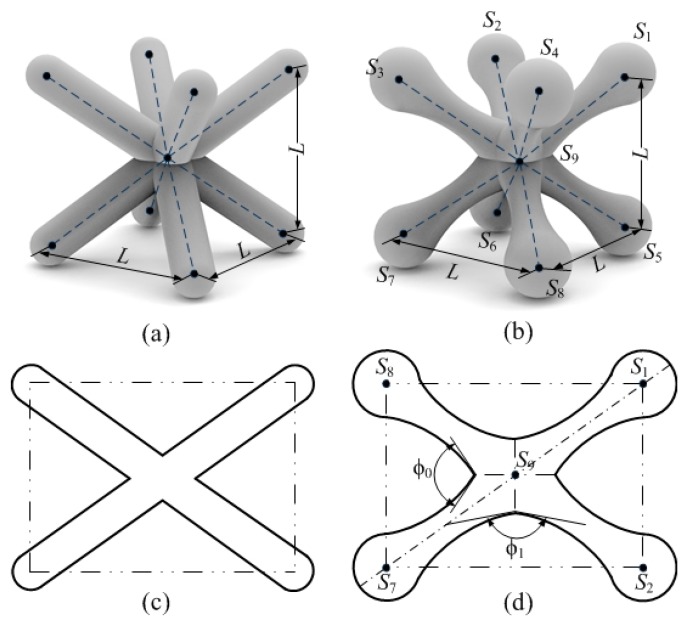
(**a**) 3D model of the BCC unit cell, (**b**) 3D model of the GBCC unit cell, (**c**) the schematic of the BCC unit cell sections, (**d**) the schematic of the GBCC unit cell sections.

The intersection of various struts forms the nodes in a unit cell; at each particular node, four sharp corners exist because of the intersection of the two struts. To reduce the stress concentration of the nodes, we increase the radii corner at the intersection of the two struts. Figure 1a,b shows the 3D model of the BCC and GBCC unit cell. Figure 1c,d depicts the schematic of the BCC and GBCC unit cell sections. To relieve the sharp corners at the node of the uniform strut, a graded-density strut with a thin midpoint and the thick end is used to replace the uniform strut. Then, the radii corner *ϕ*_0_ and *ϕ*_1_ at the node is increased to effectively reduce the stress concentration at the node. 

### 2.2. Graded-density Strut Design 

**Figure 2:**
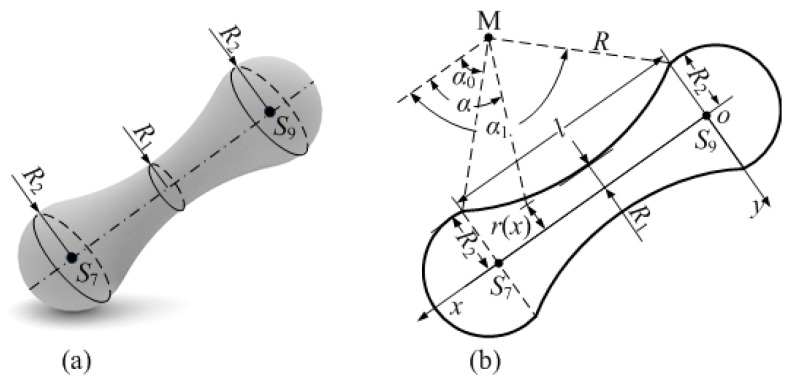
Schematic of the GBCC unit cell strut and cross section: (**a**) GBCC unit cell strut, (**b**) cross-sectional dimensions of a graded-density strut.

To ensure a uniform change of the cross section of the strut, the radial section radius of the graded-density strut gradually increases from the middle point of the strut to the two ends, where the radii of the cross-section at the midpoint and ends are *R*_1_ and *R*_2,_ respectively, as shown in Figure 2a. In the axial section of the strut, the connection line between the two ends and the middle point is a circular arc with the radius *R*, which can be determined by *R*_1_ and *R*_2_, as shown in Figure 2b.

In this paper, the GBCC unit cell is designed based on a cube with length *L*. All struts in the unit cell have identical sizes. Therefore, the strut *l_S_*_7*S*9_ is taken as an example for analysis. Figure 2b shows the cross-sectional dimensions of the graded-density strut; the radius of the graded-density strut is controlled to change uniformly by using a circular arc with the radius *R* and the span length *l,* which is equal to the strut length. The arc radius *R* is expressed through the following geometric relation:

(1) $$R=\frac{l^{2}+4{\left(R_{2}-R_{1}\right)}^{2}}{8\left(R_{2}-R_{1}\right)}$$

where *l* = $\sqrt{3}$*L*/2. 

The plane coordinate system *O_xy_* is established as shown in Figure 2b. Then, the coordinates of the point *M* of the arc center is (*l*/2, −*R*−*R*_1_), and the coordinates of any point on the arc are consistent with the following relation:

(2) $${\left(x-\frac{l}{2}\right)}^{2}+{\left[y+\left(R+R_{1}\right)\right]}^{2}=R^{2}$$

The radius *r*(*x*) of the graded-density strut in the arbitrary cross-section of the plane coordinate system *O_xy_* is as follows:

(3) $$r(x)=\left(R+R_{1}\right)-\sqrt{R^{2}-{\left(x-\frac{l}{2}\right)}^{2}}$$

The range of angle *α* between the vector *M* to any point on the arc and the *x*-axis is from *α*_0_ to *α*_1_, where *α*_1_ = π − *α*_0_. According to the theorem and geometric relations of right triangles, *α*_0_ can be obtained as 

(4) $$\alpha_{0}=\arcsin\frac{R+R_{1}-R_{2}}{R}$$

## 3. Establishment of GBCC Theoretical Model 

### 3.1. Analysis of the Relative Density of the GBCC Lattice Structure

The relative density of the lattice structure is the ratio of the volume of the lattice core to the solid core with identical thickness. The relative density is the general method to describe all lattice structure, and it is one of the key factors that affect the mechanical properties. Since the entire sandwich structure consists of many periodically distributed unit cells, the relative density of the unit cell equals the relative density of the entire structure. Therefore, only the unit cell of the lattice structure is analyzed in this paper.

**Figure 3:**
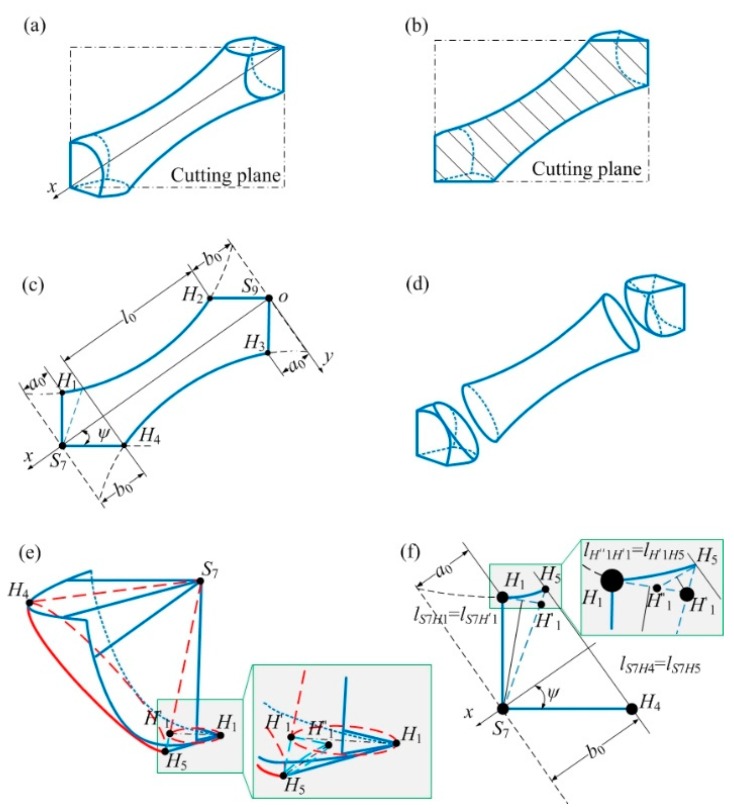
Schematic of the strut with boundary conditions of the GBCC graded-strut unit cell: (**a**) strut with boundary conditions; (**b**) cutaway view of the strut; (**c**) section size of the strut; (**d**) decomposition diagram of the graded strut; (**e**) equivalent schematic of both ends of the strut; (**f**) schematic of the equivalent part size of the strut.

The graded-density strut in Figure 3a is cut along the surface *S*_1_*S*_3_*S*_7_*S*_5_. The cutting section is shown in Figure 3b, and the size is shown in Figure 3c. To more precisely calculate the volume of the graded-density strut, the strut is cut into three parts, as shown in Figure 3d. The middle part is a graded-density strut with a length of *l*_0_ without boundary conditions, and the two ends are treated equivalently to a central cone and two small cones at each vertex. Figure 3e shows only the central cone *S*_7_-*H*_4_*H*_5_ and cone *S*_7_-*H′*_1_*H*_1_, *H′*_1_-*H″*_1_*H*_5_ at the vertex *H*_1_. Figure 3f shows a schematic of the cone *S*_7_-*H′*_1_*H*_1_, *H′*_1_-*H″*_1_*H*_5_ at vertex *H*_1_.

When the calculation is based on a strut with no boundary conditions (as shown in Figure 1b), the overlap of the graded-density struts at the unit cell node will cause the materials to stack, thereby reducing the calculation precision of the relative density. Therefore, it is necessary to perform a calculation based on the struts with boundary constraints (Figure 3a).

Parameters *a*_0_ and *b*_0_ in Figure 3 can be obtained by Equation (5) and the Pythagoras theorem as follows:

(5) $$\begin{array}{l} a_{0}=\frac{K_{3}-\sqrt{K_{3}{}^{2}+4\left(\cot^{2}\psi +1\right)K_{1}}}{2\left(\cot^{2}\psi +1\right)}\\ b_{0}=\frac{K_{2}-\sqrt{K_{2}{}^{2}+4\left(\tan^{2}\psi +1\right)K_{1}}}{2\left(\tan^{2}\psi +1\right)} \end{array}$$

where *K*_1_, *K*_2_, and *K*_3_ are constants: *K*_1_ = *R*^2^ − *l*^2^/4 − (*R + R*_1_)^2^, *K*_2_ = 2(*R + R*_1_)tan*ψ* + *l*, *K*_3_ = 2(*R + R*_1_)cot*ψ* + *l*, cot*ψ* = $\sqrt{2}\;\;$; tan*ψ* = 1/ $\sqrt{2}\;$.

The volume *V*_1_ of the central cone *S*_7_-*H*_4_*H*_5_, the volume *V*_2_ of the cone *S*_7_-*H′*_1_*H*_1_ and the volume *V*_3_ of the cone *H′*_1_-*H″*_1_*H*_5_ are obtained as

(6) $$\begin{array}{l} V_{1}=\frac{1}{3}\pi b_{0}{}^{3}\tan^{2}\psi \\ V_{2}=\frac{\pi a_{0}{}^{3}\cos2\psi }{6\sin^{3}\psi }\sin\left(\frac{\pi }{4}-\psi \right)\\ V_{3}=\frac{\pi }{6}\cos\left(\frac{\pi }{8}+\frac{\psi }{2}\right)\sin\left(\frac{\pi }{4}+\psi \right){\left(\frac{b_{0}}{\cos\psi }-\frac{a_{0}}{\sin\psi }\right)}^{3} \end{array}$$

Thus, the volume *V_l_* of the unit cell is obtained as

(7) $$V_{l}=8\left(\int_{0}^{l_{0}}\pi r^{2}\left(x\right)dx+2V_{1}+6V_{2}+6V_{3}\right)$$

The volume *V_all_* of the solid block is obtained as

(8) $$V_{all}=L^{3}$$

Therefore, for the GBCC continuous lattice structure, the relative density *ρ*′ is

(9) $$\rho^{\prime }=\frac{8\int_{0}^{l_{0}}\pi r^{2}\left(x\right)dx}{L^{3}}+\frac{16\left(V_{1}+3V_{2}+3V_{3}\right)}{L^{3}}$$

**Figure 4:**
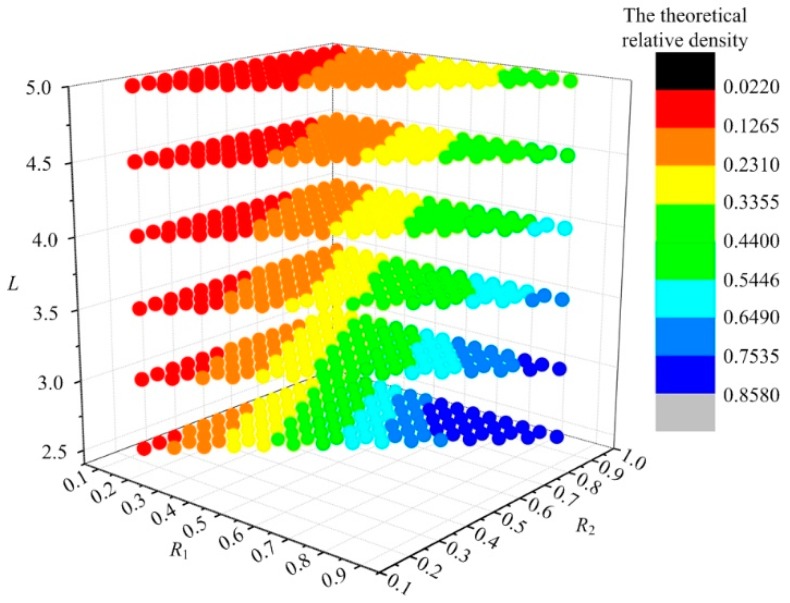
Diagram of the relationship between theoretical relative density and *R*_1_, *R*_2_, *L*.

The relationship between *R*_1_, *R*_2_, *L* and theoretical relative density is shown in Figure 4. For the same *L*, the theoretical relative density is positively correlated with *R*_1_ and *R*_2_. For the same *R*_1_ or *R*_2_, the theoretical relative density has a negative correlation with *L* and a positive correlation with *R*_2_ or *R*_1_. The maximum value of the theoretical relative density occurs where *L* is the smallest and *R*_1_, *R*_2_ are the largest. In addition, different combinations of *R*_1_, *R*_2_ and *L* will have the same relative density. Therefore, the relative density of lattice structure can be made equal by changing the value of *R*_1_, *R*_2_ and *L*, compared to the mechanical properties. The establishment of the relationship between *R*_1_, *R*_2_, *L* and theoretical relative density provides guidance for the determination of parameters of lattice structure sample.

### 3.2. Force Analysis of the GBCC Lattice Structure 

The force condition of the lattice unit cell largely affects the lattice structure performance of mechanical properties such as the initial stiffness and the plastic failure strength. Therefore, it is necessary for the theoretical analysis of the unit cell to establish a mechanical model.

Referring to a previous report [12], the following assumptions can be made:

1. All struts in a unit cell are slender struts, idealized as Euler-Bernoulli beams;
2. The deformation of the strut is always on the diagonal surface of the unit cell with the strut axis. For example, the deformation of struts *S*_1_*S*_7_ and *S*_3_*S*_5_ is in the diagonal plane *S*_1_*S*_3_*S*_7_*S*_5_.

According to the above hypothesis, the strut in the unit cell can be considered a flat beam element. The beam element is subjected to the axial force, the shear force and the bending moment. The beam element is in a combined deformation state of axial compression and plane bending without rotation. For the GBCC lattice structure, each strut can be used as a unit for the mathematical analysis.

**Figure 5:**
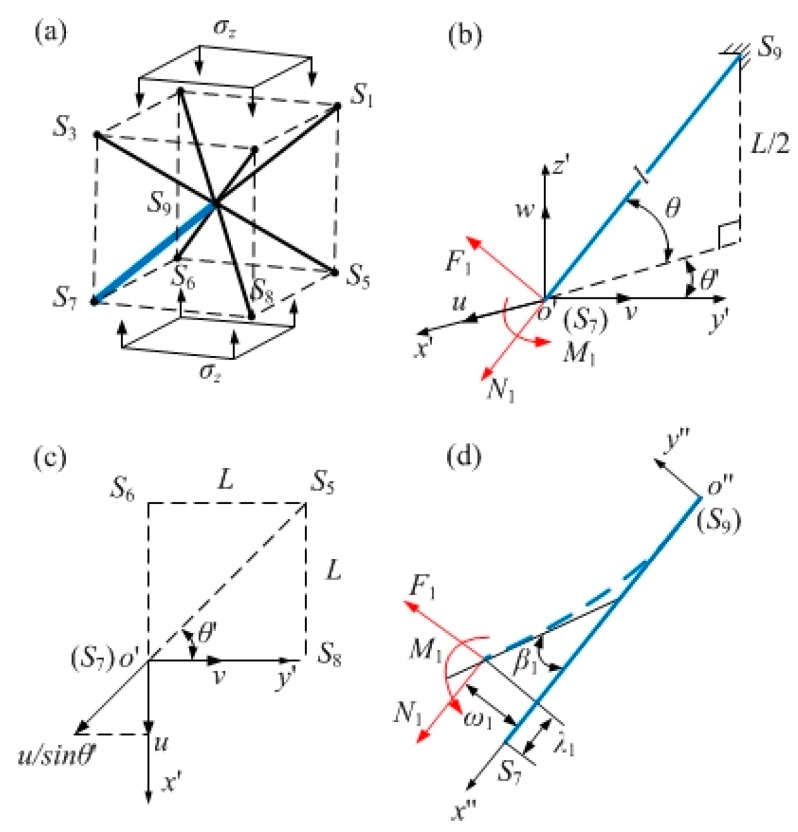
Schematic of the GBCC unit cell and strut under force: (**a**) unit cell under force; (**b**) space force and deformation of the strut; (**c**) displacement of *S*_7_ in the plane coordinate system *o*′*x*′*y*′; (**d**) force and deformation of the strut in the plane coordinate system *o*″*x*″*y*″.

As shown in Figure 5a, when the GBCC unit cell is subjected to a compressive stress *σ_z_*, the node can move arbitrarily in space. Since the entire unit cell is symmetric about point *S*_9_, each strut force will cancel each other at *S*_9_, so the node displacement at *S*_9_ is zero. Taking strut *S*_7_*S*_9_, for example, the angle between the strut *S*_7_*S*_9_ and the plane *S*_5_*S*_6_*S*_7_*S*_8_ is *θ*, and the angle between *S*_5_*S*_7_ and *S*_7_*S*_8_ is *θ*′. The displacements of *S*_7_ in the space coordinate system *O*′*_x_*_′*y*′*z*′_ are (*u*, *ν*, *w*) for the action of an axial force *N*_1_, the tangential force *F*_1_ and the moment *M*_1_, as shown in Figure 5b,c.

The deformation schematic of the GBCC unit strut is shown in Figure 5d; with the fixed point *S*_9_ of the equivalent cantilever beam as the origin, the plane coordinate system *O*′′*_x_*_′′*y*__′′_ is established. The plane axial displacement, the deflection and the section angle of the strut are *λ*_1_, *ω*_1_, and *β*_1_, respectively, which are generated by the axial force *N*_1_, the tangential force *F*_1_ and the bending moment *M*_1_. The solid line is the initial state of the strut, and the dotted line is the state after deformation.

Based on the above analysis, the axial displacement of the strut can be obtained from displacement (*u, ν, w*) in the space coordinate system *o*′*x*′*y*′*z*′*:*

(10) $$\lambda_{1}=\frac{2u-w}{\sqrt{3}}$$

According to Hooke’s law, the axial displacement of the strut under the action of axial force *N*_1_ is

(11) $$\lambda_{1}=\frac{RN_{1}C_{1}}{\pi E_{s}}$$

In this paper, *B_i_* = *B_i_*(*α*) is a function of *α*, and *C*_i_ is a constant variable. In Equation (11), *E_s_* is the elastic modulus of the parent material, *B*_1_(*α*) = sin*α*/[*R*sin*α* − (*R* + *R*_1_)]^2^, and $C_{1}=\int_{\alpha_{0}}^{\alpha_{1}}B_{1}d\alpha$. 

Combined with Equations (10) and (11), the axial force *N*_1_ is

(12) $$N_{1}=\frac{\pi E_{s}\left(2u-w\right)}{\sqrt{3}RC_{1}}$$

The tangential displacement of the strut can be obtained from the displacement (*u*, *ν*, *w*) in the space coordinate system *o*′*x*′*y*′*z*′:

(13) $$\omega_{1}=\frac{\sqrt{6}}{3}\left(u+w\right)$$

In the plane coordinate system *o*″*x*″*y*″, since the strut is affected by both the tangential force *F*_1_ and the bending moment *M*_1_, according to the structural combined deformation of bending and compression, the tangential displacement of the strut under the action of the tangential force and the bending moment is

(14) $$\omega_{1}=\frac{4R\left(F_{1}C_{2}-M_{1}C_{3}\right)}{\pi E_{s}}$$

where *B*_2_(*α*) = sin*α*/[*R*sin*α* − (*R* + *R*_1_)]^4^, *B*_3_(*α*) = *R*cos*α* + *L*/4, $C_{2}=\int_{\alpha_{0}}^{\alpha_{1}}B_{2}B_{3}^{2}d\alpha$, $C_{3}=\int_{\alpha_{0}}^{\alpha_{1}}B_{2}B_{3}d\alpha$.

In addition, since the lattice structure is a central symmetric structure, the bending moment at each node is identical. Thus, the bending moments *M_S_*_7_ and *M_S_*_9_ at *S*_7_ and *S*_9_ are identical and expressed as *M*_1_ [12]:

(15) $$M_{1}=\frac{F_{1}l}{2}$$

In combination with Equations (13)–(15), the tangential force *F*_1_ and the bending moment *M*_1_ are

(16) $$\left\{ \begin{array}{l} F_{1}=\frac{2\pi E_{s}\left(u+w\right)}{RC_{4}}\\ M_{1}=\frac{\sqrt{2}\pi E_{s}L\left(u+w\right)}{4RC_{5}} \end{array}\right.$$

where $\begin{array}{l} C_{4}=\int_{\alpha_{0}}^{\alpha_{1}}B_{2}B_{3}\left(4\sqrt{6}B_{3}-3\sqrt{2}L\right)d\alpha \\ C_{5}=\int_{\alpha_{0}}^{\alpha_{1}}B_{2}B_{3}\left(4B_{3}-\sqrt{3}L\right)d\alpha \end{array}$

According to the tangential force *F*_1_ and the bending moment *M*_1_ of *S*_7_ in the space coordinate system *o*′*x*′*y*′*z*′, the force *F_z_*_′_ in the *z*′ direction is

(17) $$F_{z'}=F_{1}\cos\theta -N_{1}\sin\theta$$

where cos*θ* = $\sqrt{6}\;\;$/3 and sin*θ* = 1/$\sqrt{3}\;$.

In addition, because the endpoints *S*_5_, *S*_6_, *S*_7_ and *S*_8_ of the four struts at the bottom of the unit cell are equally affected by the pressure stress *σ_z_*, the *z*′ direction force *F_z_*_′_ of each endpoint is *σ_z_L*^2^/4.

The deformations of the strut are mainly the axial deformation and the transverse bending deformation, and its elastic strain energy includes both the axial expansion energy and the bending strain energy. Based on the work-energy principle (the strain energy of the lattice structure is the work done by the external force and moment on the structure) and Hooke’s law, the strain energy *U* of the *S*_7_*S*_9_ strut is affected by the axial force *N*_1_, the tangential force *F*_1_ and moment *M*_1_, as shown below:

(18) $$U=\frac{R\left(N_{1}{}^{2}C_{1}-M_{1}F_{1}C_{6}+F_{1}{}^{2}C_{7}\right)}{2\pi E_{s}}$$

where B_4_(α) = 2Rcosα + l, B_5_(α) = B_4_^2^(α) + l^2^, $C_{6}=4\int_{\alpha_{0}}^{\alpha_{1}}B_{2}B_{4}d\alpha$, $C_{7}=\int_{\alpha_{0}}^{\alpha_{1}}B_{2}B_{5}d\alpha$.

There are eight struts in each unit cell, therefore, *U_GBCC_* = 8*U*. 

The work *U_w_* done by the compressive stress *σ_z_* on the GBCC unit cell in the *z*′ direction is

(19) $$U_{w}=2\sigma_{z}L^{2}w$$

According to the energy conservation law, the strain energy of a unit cell is equal to the work done by the external force, i.e., *U_GBCC_ = U_w_*. Combining Equations (18) and (19), the following equation is obtained:

(20) $$L^{2}\sigma_{z}w=\frac{2R\left(N_{1}{}^{2}C_{1}-M_{1}F_{1}C_{6}+F_{1}{}^{2}C_{7}\right)}{\pi E_{s}}$$

Combining Equations (12) and (16), and changing the equation for *F*_1_ to:

(21) $$F_{1}=\frac{2RlN_{1}C_{1}+3\pi E_{s}Lw}{RLC_{4}}$$

The equation for *N*_1_ can be obtained from Equation (17) and *F_z′_* = *σ_z_L*^2^/4.

(22) $$N_{1}=\frac{4F_{1}\cos\theta -\sigma_{Z}L^{2}}{4\sin\theta }$$

In combination with Equations (15), and (20)–(22), *u* and *ω* can be eliminated to obtain the quadratic equation of *F*_1_:

(23) $$C_{9}F_{1}{}^{2}+C_{10}F_{1}+C_{11}=0$$

where

(24) $$\left\{ \begin{array}{l} C_{8}=\int_{\alpha_{0}}^{\alpha_{1}}B_{2}\cos^{2}\alpha d\alpha \\ C_{9}=-2R\left(2R^{2}C_{8}+C_{1}\right)\\ C_{10}=\frac{R\sigma_{z}L^{2}\left(C_{5}+2C_{1}\right)}{\sqrt{6}}\\ C_{11}=-\frac{R\sigma_{z}{}^{2}L^{4}C_{1}}{16} \end{array}\right.$$

With Equation (23), the tangent force *F*_1_ is obtained:

(25) $$F_{1}=\frac{\sqrt{6}L^{2}\sigma_{z}C_{13}}{8C_{12}}$$

where $\begin{array}{l} C_{12}=2R^{2}C_{8}+C_{1}\\ C_{13}=\frac{C_{5}+2C_{1}}{3}+\sqrt{\frac{{\left(C_{5}+2C_{1}\right)}^{2}}{9}-\frac{C_{12}C_{1}}{3}} \end{array}$

According to Equations (15), (22) and (25), the axial force *N*_1_ and the bending moment *M*_1_ are:

(26) $$\begin{array}{l} N_{1}=\frac{\sqrt{3}L^{2}\sigma_{z}\left(C_{13}-C_{12}\right)}{4C_{12}}\\ M_{1}=\frac{3\sqrt{2}L^{3}\sigma_{z}C_{13}}{32C_{12}} \end{array}$$

To verify the correctness of Equations (25) and (26), this paper makes comparative analysis with FEA, the prediction bias *ξ*_1_ (as shown in Equation (27)) of the theoretical model of the GBCC unit cell under the action of a certain stress *σ_z_* is listed in Table 1. Since *F*_1_, *N*_1_ and *M*_1_ can be derived from each other, the table only compares the value of *M*_1_.

(27) $$\xi_{1}=\frac{\left|M_{1}\left(\mathrm{Theory}\right)-M_{1}\left(\mathrm{FEA}\right)\right|}{M_{1}\left(\mathrm{FEA}\right)}$$

where *M*_1_ (Theory) and *M*_1_ (FEA) refer to the theoretical and finite element simulation values respectively.

The struts of the lattice unit cell must meet certain requirements: adjacent struts can not intersect with each other, the slenderness must be greater than the ultimate slenderness and have a high fabrication accuracy, and the strut radius should be in the range 0.15 mm–0.9 mm [16]. At the same time, in order to control the variables of *R*_1_, *R*_2_, *L* and to change the stress in a wide range, five groups of samples are selected. It can be seen that the theoretical model has a good prediction accuracy for the selected groups of samples, which proves the correctness of the theoretical model. In addition, as the equivalent aspect ratio of the unit cell (the aspect ratio of the BCC equivalent model that has the same unit cell length and relative density as the GBCC unit cell) becomes larger, the prediction accuracy becomes better.

## 4. Experimental and Finite Element Simulation Details 

Three groups of samples were selected for finite element simulation and experiment. In the same group, all samples had the same relative density, and different combinations of *R*_1_ and *R*_2_ made the struts have different contour curves, thus making the nodes have a different radii corner. Each group of samples contained three GBCC samples and one BCC sample. The relevant sample parameters are listed in Table 2. The number of the sample is composed of the group number of the sample and its number in the group. The sample size (length, width and height) is 32 mm, 32 mm and 16 mm. *ρ*′ is the relative density of the 3D unit cell. In this paper, Ti6Al4V is used as the manufacturing material, and the lattice structure samples are fabricated by the SLM method. The fabrication quality of the samples is analyzed using a field emission scanning electron microscope. The uniaxial quasi-static compression experiment was performed with a universal material testing machine.

### 4.1. Finite Element Analysis of the Lattice Structure 

The finite element simulation software used for quasi-static uniaxial compression of lattice structure is Abaqus/Explicit 6.14-2. The density, elastic modulus and Poisson’s ratio of the materials used in the simulation are 4.43 × 10^−9^ Tonne/mm^3^, 11,8000 MPa and 0.3, respectively [47]. To further observe the stress state and the failure mode of the lattice structure during the quasi-static compression, the constitutive models and the failure model of the lattice structure are added to the material setting. 

The constitutive model chosen in this paper is the Johnson-Cook (J-C) phenomenological constitutive model. This model considers the separated effects of the strain hardening, the strain-rate (viscosity) and the thermal softening [48], as shown in the following equation:

(28) $$\bar{\sigma }=g\times h\times k$$

where $g=A+B{\bar{\varepsilon }}^{n}$ indicates the strain hardening effect; $h=1+C\ln\left(\dot{\bar{\varepsilon }}/\dot{\bar{\varepsilon_{o}}}\right)$ refers to the strain-rate (viscosity) effect; $k=1-{\left[\left(T-T_{room}\right)/\left(T_{m}-T_{room}\right)\right]}^{m}$ shows the thermal softening effect; $\bar{\sigma }$ is the equivalent plastic stress (MPa); $\bar{\varepsilon }$ is the equivalent plastic strain, $\dot{\bar{\varepsilon }}$ is the equivalent plastic strain rate (S^−1^); $\dot{\bar{\varepsilon_{o}}}$ is the reference equivalent plastic strain rate (S^−1^); *T* is the temperature (°C), *T_m_* is the melting temperature of the work material (°C) and *T*_room_ is the room temperature (°C). *A*, *B*, *C*, *m* and *n* are material parameters.

The selected fracture-based model is the J-C ductile fracture model, as this model considers both the stress triaxiality and the strain-rate effects [48]. This model is usually represented by the following equation:

(29) $${\bar{\varepsilon }}_{f}=a\times b\times c$$

where $a=d_{1}+d_{2}\exp\left(d_{3}\eta \right)$; $b=1+d_{4}\ln\left(\dot{\bar{\varepsilon }}/\dot{\bar{\varepsilon_{o}}}\right)$; $c=1+d_{5}\left[\left(T-T_{room}\right)/\left(T_{m}-T_{room}\right)\right]$ ${\overset{\text{¯}}{\varepsilon }}_{f}$ is the equivalent fracture strain; η=Pσ¯ is the stress triaxiality parameter. The determination of the five parameters (*d*_1_…*d*_5_) involves a series of experimental fracture tests by varying the stress triaxiality, the strain-rate and the temperature [48]. The parameters used in the J-C constitutive model and the J-C ductile fracture model are shown in Table 3 [48].

The failure displacement is set to 0.004. As the crushing mechanism of the lattices is a very complex process and involves all three types of nonlinearities, it can not be simulated with implicit solvers [49]. In addition, for quasi-static simulations, ABAQUS/Explicit saves computing resources and computation time over ABAQUS/Standard, so this simulation uses the ABAQUS/Explicit solution method.

**Figure 6:**
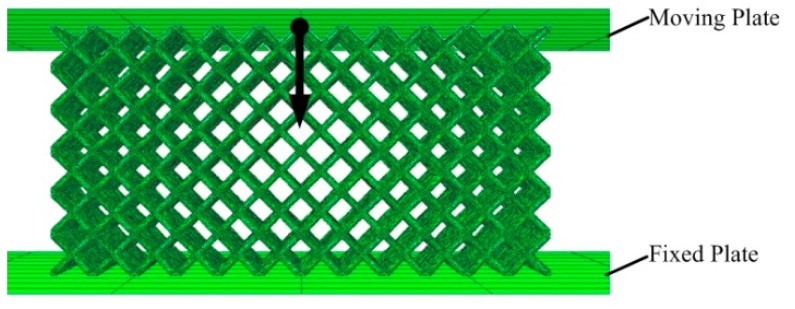
Boundary conditions of a lattice structure sample.

The element size of the lattice structures is set to 0.8 mm and the mesh is a tetrahedral mesh. As for boundary conditions of structures, two rigid plates were placed in the height direction of the lattice block, as shown in Figure 6. The surface accuracy of the indenter is high, the contact area is small, and the tangential load is not applied. So, the friction between the indenter and the sample can be neglected. Therefore, in the simulation setup, tangential friction-free contact is adopted and there is no frictional contact between the rigid plate and the lattice block. The moving plate has only one degree of freedom, moving downwards at a small constant speed in the direction of the arrow shown at a distance of 5 mm, and the freedom of the fixed plate is zero. When the whole process is finished, the stress-strain curve and failure mode of the lattice structure under quasi-static compression is obtained.

### 4.2. Sample Fabrication

The fabrication equipment is the British Renishaw AM250 laser melting and rapid prototyping machine (United Kingdom), and the fabrication parameters are as shown in Table 4. The raw material is Ti6Al4V titanium alloy powder. The molding method is line scanning. The fabrication direction of lattice structure is the compression direction of structure in quasi-static uniaxial compression experiment, ensuring that the lattice structure has better mechanical properties.

The emission scanning electron microscope used in this paper is the TESCAN VEGA 3 LMH SEM (Brno, Czech Republic). The uniaxial quasi-static compression tests of samples were performed by using the SANS electro-mechanical universal testing machine of the MTS company. At room temperature 25 °C, the sample was placed on the horizontal bench of the universal material testing machine. The test machine compressed the sample at a speed of 1 mm/min and recorded the load-displacement data.

**Figure 7:**
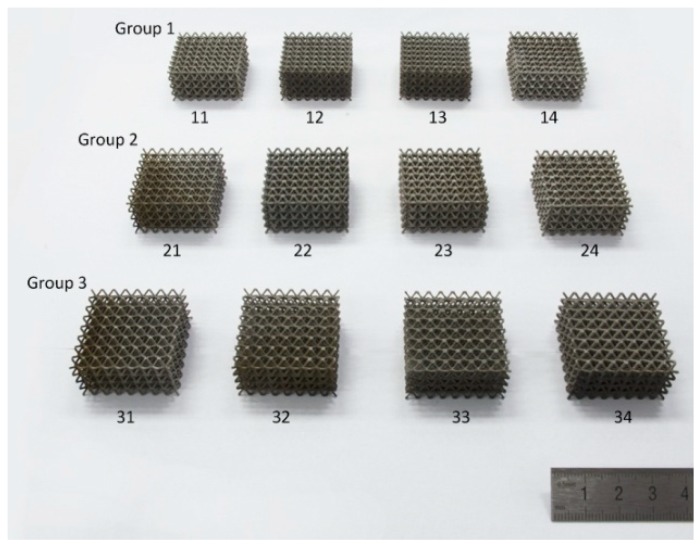
Samples fabricated by SLM.

**Figure 8:**
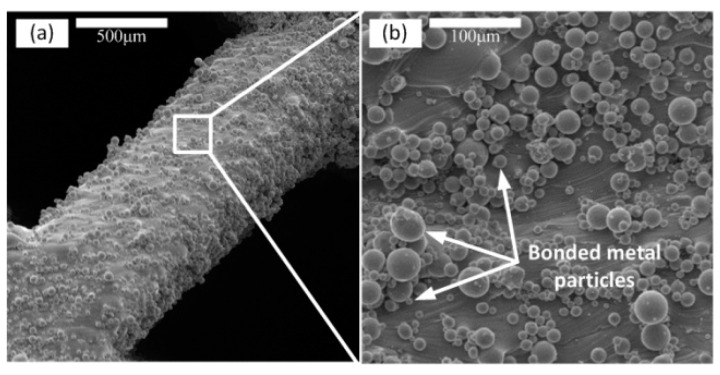
Surface topography of unit cell strut, (**a**) unit cell strut, (**b**) partial enlargement of unit cell strut (×300).

**Figure 9:**
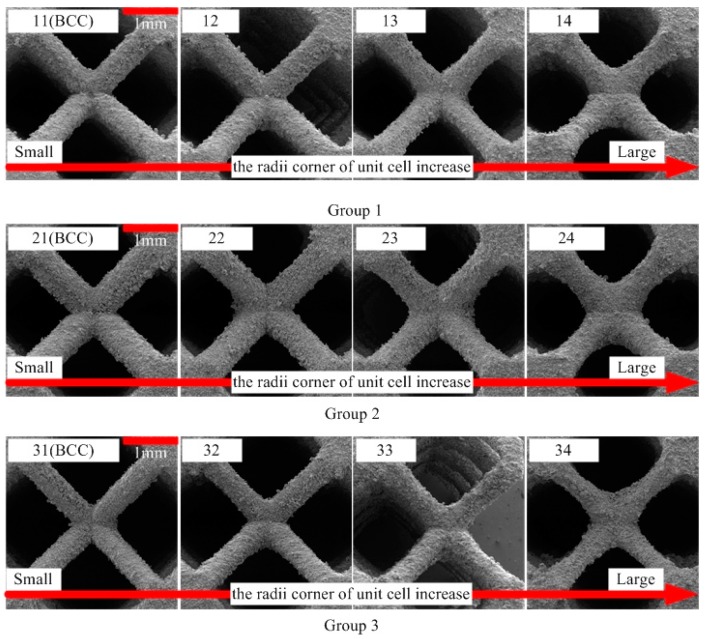
The electron micrographs of the sample unit cells.

The finished samples are shown in Figure 7. Figure 8 shows the surface topography of the unit cell strut in samples. A higher magnification SEM micrograph of the strut demonstrates many partially melted metal particles bonded on the surfaces of the lattice structures. The electron micrograph of the sample unit cell is shown in Figure 9.

The rough strut surfaces of the SLM-manufactured lattice structures can be attributed to two reasons: (1) in the SLM process, the circular struts are fabricated by melting and solidifying the loose metal powder layer by layer, and the partially molten or semi-molten metal powders of each preparation layer will adhere to each preparation layer and its edges. (2) Thermal diffusion occurs between loose powder and solid material because of large temperature difference, causing the powder particles to stick to the strut surface [50]. However, the rough strut surfaces of a unit cell do not significantly affect the compressive properties of the structure [51,52].

## 5. Results and Discussion

### 5.1. Deformation of GBCC Lattice Structures Subsection

**Figure 10:**
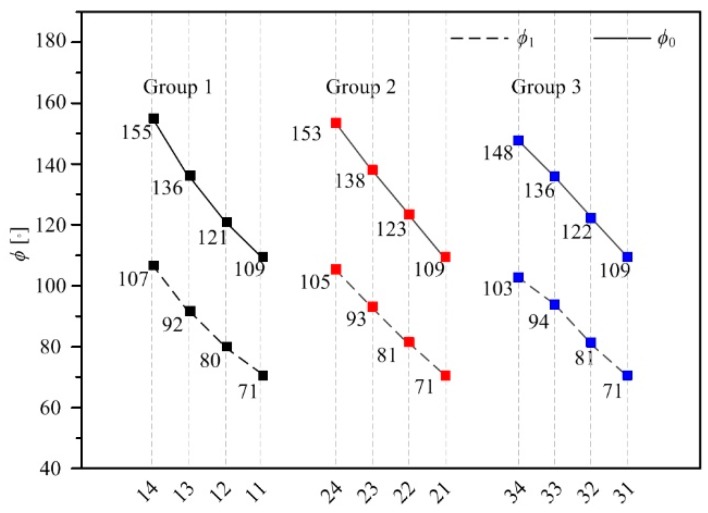
The *ϕ*_0_ and *ϕ*_1_ values of samples.

The radii corner *ϕ*_0_ and *ϕ*_1_ of the unit cell nodes corresponding to samples with different structural parameters are shown in Figure 10. For lattice structure groups with the same relative density and cell side length, the gradual change of radii corner of the unit cell is realized by gradually changing the *R*_1_ and *R*_2_ values. The *ϕ*_0_ and *ϕ*_1_ are larger than BCC for all GBCC samples, indicating that this design method of GBCC lattice structure can indeed increase the radii corner at the intersection of the two struts. By studying the differences in mechanical properties and failure mode of several groups of samples, the relationship between the radii corner at the intersection of the two struts can be studied further, as well as the mechanical properties and failure mode.

**Figure 11:**
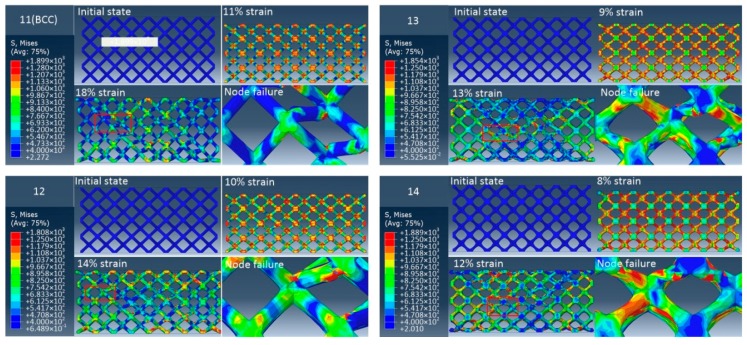
The FEA failure process diagram of Group 1.

**Figure 12:**
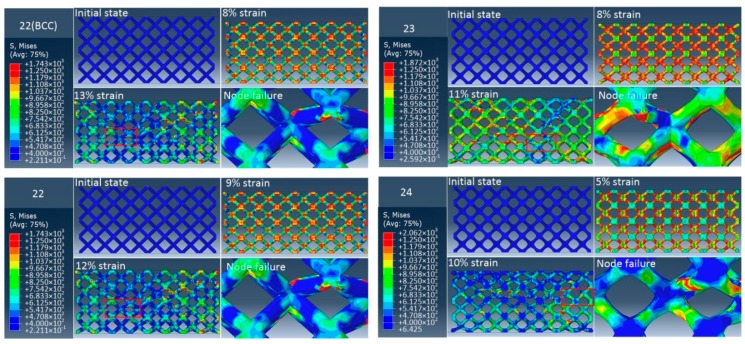
The FEA failure process diagram of Group 2.

**Figure 13:**
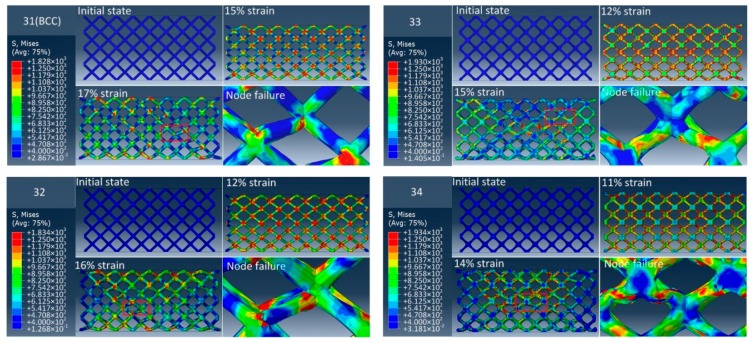
The FEA failure process diagram of Group 3.

To compare the elimination effects of stress concentration at the node of unit cell with different radii corners, quasi-static uniaxial compression experiments were carried out on samples. Figure 11, Figure 12 and Figure 13 show the stress diagram and failure mode of three groups of samples in the quasi-static compression simulation using the finite element method. It can be observed that the stress of the BCC lattice structure is mainly concentrated at the node, and its failure mode is that the strut is completely broken from the node. For a group of samples with the same relative density, with the increase of the radii corner of unit cell nodes, the stress concentration area will no longer concentrate completely at the node, but gradually move towards the middle of the strut. Also, the fracture position of the strut will gradually move away from the node. This shows that the method of increasing the radii corner of unit cell nodes can significantly relieve the stress concentration effects at the node. At the same time, it is noticed that with the increase of the radii corner of unit cell nodes, the failure displacement of the structure will become smaller; that is, the ductility of the structure is getting worse. For structures with different relative density, with the increase of relative density, the structure failure displacement decreases and the ductility decreases. In addition, the failure modes of all samples are mainly 45-degree failure modes, accompanied by layer-by-layer crushing failure modes.

**Figure 14:**
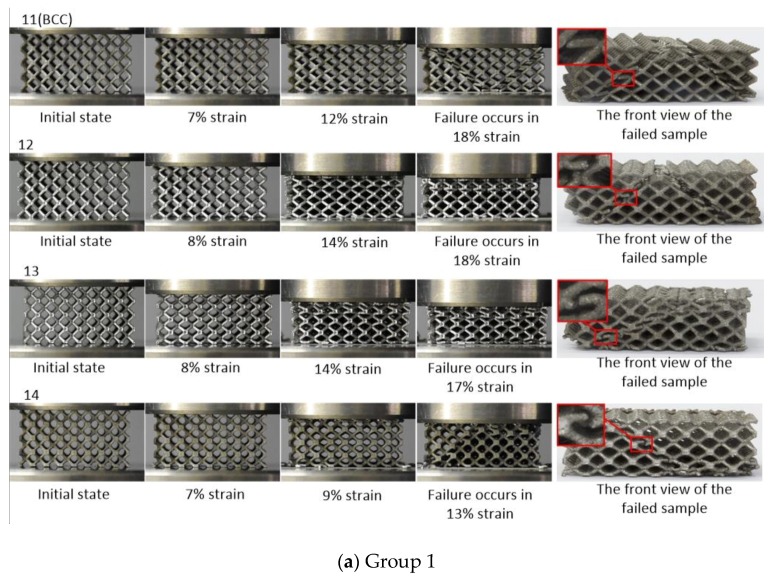

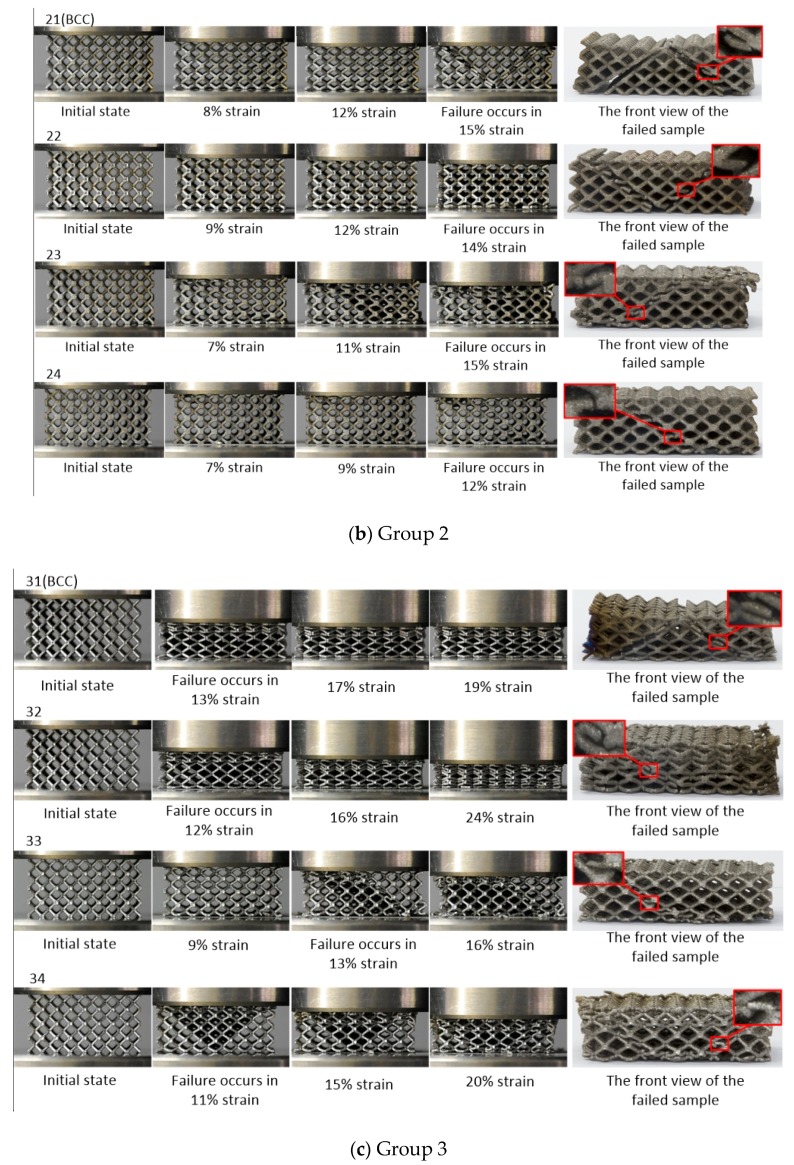
The sample failure process diagram of (**a**) (**b**) and (**c**).

Figure 14 shows the deformation modes of the GBCC and BCC lattice structure samples from the initial state to the failure state in the uniaxial compression test. Similar to the FEA results, for the same relative density sample and with the increase of the radii corner of unit cell nodes, the damage position of the strut gradually moves away from the node and the ductility of the structure gradually becomes worse. In addition, for different relative densities, it should be noted that with the increase of the relative density of the structure, the failure mode gradually changes from the mixed failure mode of layer-by-layer failure and 45-degree failure to the failure mode dominated by 45 degrees damage. Group 3 is a group of samples with the lowest relative density. The failure modes of the structure include 45-degree failure and layer-by-layer failure mode. Group 2 is a group of samples with the highest relative density, and its dominated failure mode is the 45-degree failure, with the lattice structure slipping on both sides of the shear zone. So, it can be seen from the above that the design method of increasing the radii corner of unit cell nodes can improve the stress concentration effect of the nodes.

As can be seen from Figure 14. In the compression process, the middle part of the sample exhibits slight elastic expansion towards the surrounding area. The reasons for this phenomenon are as follows: 1) the internal deformation of the sample is not uniform; 2) frictional force exists between the platform of the test machine and the top and bottom of the sample, rendering the top and bottom parts of the sample unable to extend to the surrounding area. Also, the middle part of the sample can expand to the surrounding area because of the lack of boundary conditions. However, it can be seen from the previous part of this paper that the frictional force is so small that it can be neglected.

### 5.2. Relative Density

The relative density is one of the key factors that affect the mechanical properties of lattice structures. To compare the mechanical properties of lattice structure, the samples must have identical relative density. Table 5 shows the relative density of the fabricated samples. The relative density deviation of the same group is within 9%, indicating that the samples have high fabrication accuracy and the comparison of each group of structures has the same basis. The theoretical prediction bias *ξ* for the relative density of each sample is shown in Equation (30).

(30) $$\xi =\frac{\left|\rho \left(\mathrm{Theory}\right)-\rho \left(\mathrm{SLM}\right)\right|}{\rho \left(\mathrm{SLM}\right)}$$

where *ρ*(Theory) and *ρ*(SLM) refer to the theoretical calculation value and the fabricated sample value, respectively.

For all the samples to be researched, *ξ* is not higher than 9%, which proves the correctness of the theoretical calculation method for the relative density of structures.

### 5.3. Mechanical Properties of GBCC Lattice Structure

#### 5.3.1. Mechanical Response of Experiments and Finite Element Simulation

**Figure 15:**
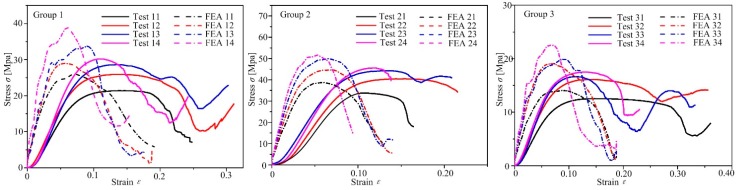
Stress-strain curve of three groups of samples.

The stress-strain curves of three groups of samples in the finite element simulation and the quasi-static uniaxial compression tests are shown in Figure 15. It can be noticed that there are some deviations between the FEA curve and the experimental data fitting curve. The reasons are as follows: (1) in the SLM process, partially melted powder adheres to the surface of the strut, so its forming diameter and design diameter will vary; (2) rapid melting and cooling of the powder can cause residual stresses in the structure. Due to the influence of processing technology, there are differences between the sample strut diameter and the designed strut diameter, which lead to deviation in pore size, strut diameter and porosity between the 3D models and the fabricated samples. The variation, caused by SLM processing, of structural size parameters and mechanical parameters has been analyzed, and if the change of structure size is considered, the prediction accuracy of sample size and performance parameter value can be improved, which is verified by FEA [53]. In general, the FEA data and experimental data exhibit consistent trends.

The test curve exhibits two regimes before structural failure. The first regime is a linear region. At this time, the lattice structure is in the elastic deformation stage. However, the “linear elasticity” stage of the stress-strain curve obtained from the experiment is not actually linear, but concave downward. The reason for this phenomenon is that with the increased elastic bending deformation of the strut, the axial load of the strut correspondingly increases. When the axial load reaches the Euler load, the strut yields. In the compression process, this beam-column interaction will reduce the initial stiffness of the structure [54] and may cause the concavity. The linear fitting method was used in the elastic part to obtain the accurate experimental initial stiffness *E*. The second region is the plateau regime. Because the GBCC and BCC unit cell structure have eight struts and nine joints, it’s Maxwell number *M* [55] is 8−3×9+6=−13<0, satisfying Maxwell’s criterion for bending-dominated structures. Therefore the GBCC and BCC lattice structure are bending-dominated structures, and have a large constant extension of platform stress. The energy absorption characteristics of the lattice structure are mainly related to this stage. When the strain of samples reaches the ultimate strain, the structure begins to break. The ultimate strain corresponds to the plastic failure strength *σ*′, which is the ratio of the maximum external load to the cross-sectional area of the structure.

As the compression continues, most of the samples will reach the densification stage and the stress will increase. In the structure within the densification stage, the damaged part is not completely detached. Therefore, the unit cells that are in contact with each other will also have a certain carrying capacity.

#### 5.3.2. Initial Stiffness and Plastic Failure Strength

As can be seen from Table 6, for the same relative density, the plastic failure strength *σ*′ and the initial stiffness *E* of GBCC is much larger than the BCC lattice structure. The lower the relative density, the greater the increase in the plastic failure strength and initial stiffness of GBCC. For example, in Group 3, which has the lowest relative density, the initial stiffness of sample 32 was 27.6% higher than that of 31 (BCC), and the plastic failure strength was 30.9%; the initial stiffness of sample 12 was 19.8% higher than that of 11 (BCC), and the plastic failure strength was 22.8%; the initial stiffness of sample 22 was 15.4% higher than that of 21 (BCC), and the plastic failure strength was 18.5%. It is shown that at the same relative density, the GBCC lattice structure with larger radii corner will have better mechanical properties than the BCC ones, and the improvement of this performance is more obvious for the BCC lattice structure with smaller relative density.

By adjusting the structure parameters for each group of samples with the same relative density, the angle *ϕ*_0_ and *ϕ*_1_ of the structure will increase, and the plastic failure strength *σ*′ and the initial stiffness *E* will also increase, which means that the bearing capacity of the structure will become better. Therefore, the increment of the radii corner at the intersection of two struts can indeed improve the mechanical properties of the structure, proving the feasibility of the graded-strut design method.

In addition, it is noted that the improvement of the mechanical properties between the GBCC lattice structures is smaller than that between the GBCC and the BCC lattice structures, indicating that as the radii corner at the intersection of the two struts increases, the improvement of the stress concentration effect will also slow down. This can be explained as follows: for the same relative density, increasing the radii corner at the intersection of two struts can indeed improve the stress concentration effect and increase the strength at the node, but it will also weaken the strength of the strut.

Table 6 also shows the relationship between the relative density, the radii corner of the unit cell nodes and the mechanical properties of samples. Since both *ϕ*_0_ and *ϕ*_1_ represent the radii corner, *ϕ*_1_ is used as the independent variable only. It is noteworthy that, compared to the radii corner of the unit cell nodes, the relative density has a much greater improvement in mechanical properties, which indicates that the relative density is the most important factor that affects the mechanical properties of the unit cell. Therefore, in specific application environments, it is necessary to comprehensively consider the mechanical performance requirements and the relative density requirements of the lattice structure, thereby achieving the predetermined mechanical performance by graded-strut design method within the maximum allowable relative density range.

#### 5.3.3. Energy Analysis

**Figure 16:**
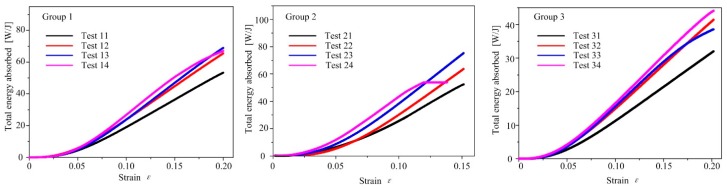
Energy analysis of the GBCC and BCC samples.

The metal lattice structure is superior to the traditional solid structure due to its excellent properties, such as low density, high specific stiffness and strength, and high energy-absorbing capability. The total energy absorbed by the experimental sample and the strain before the plastic failure of the structure is shown in Figure 16, in which there is a positive correlation between the total energy absorbed by the experimental sample and the strain. This can be explained as follows: when the load increases, plastic buckling occurs at the struts, followed by rotations of the plastic hinge. Thus, the energy absorption capacity improves [6,17,43].

Figure 16 shows that in the case of the same relative density, the GBCC samples can absorb more energy than the BCC reference samples under identical strain. For the same group of GBCC samples, as the radii corner of the unit cell nodes increased, the total absorbed energy also increased, indicating that the graded-strut design method of increasing the radii corner of the structure can improve the energy absorption performance of the structure. In addition, as the relative density of the structure increased, the upper limit of energy absorption of lattice structure also became higher.

## 6. Conclusions

In view of the stress concentration effect at the unit cell nodes of BCC lattice structure, which weakens the mechanical properties of the structure, a graded-strut design method is proposed. The theoretical calculation, finite element simulation and experimental results validate the proposed design method for the unit cell. The main conclusions are as follows:

1. To relieve the stress concentration defects at the nodes of the BCC lattice structure and improve the mechanical properties, the radii corner of the BCC unit cell nodes should be increased. A lattice structure with graded struts (namely, the GBCC structure) is proposed accordingly.
2. A general model of the GBCC lattice structure is established. By combining the classical beam theory with the energy conservation law, a specific mapping relationship between the GBCC unit cell size and the mechanical model of the lattice structure is proposed. The mathematical prediction models for the relative density of the GBCC lattice structure are also established. For all the GBCC and BCC samples, the theoretical prediction accuracy of the force model ξ_1_ is less than 16%, and the theoretical prediction accuracy of the relative density *ξ* is less than 9%, which proves the correctness of the theoretical calculation method for the force and the relative density analysis of the lattice structure.
3. Under identical experimental conditions and density parameters, the finite element simulation, the fabrication and experimental tests of the new GBCC structure and BCC reference structure were completed. The Johnson-Cook (J-C) phenomenological constitutive model and the J-C ductile fracture model are added to the finite element simulation to simulate the stress distribution of the failed lattice sample node. As the radii corner at the intersection of two struts increases, for the same relative density, the stress concentration effect at the lattice structure node is improved, and the failure position of the strut will gradually move away from the node, thereby improving the mechanical properties (including compression and energy absorption properties).

## Figures and Tables

**Table 1:** Prediction bias of GBCC unit cell theoretical force model.

*L* (mm)	*R*_1_ (mm)	*R*_2_ (mm)	*σ_z_* (MPa)	*M*_1_ (Theory) (N·mm)	*M*_1_ (FEA) (N·mm)	*ξ*_1_ (%)
4.00	0.330	0.430	17.4	194.4	174.2	11.6
4.00	0.275	0.385	11.9	132.8	134.7	1.4
4.00	0.180	0.265	3.1	34.7	34.3	1.0
3.25	0.180	0.265	5.1	30.7	34.1	9.9
2.50	0.180	0.265	5.5	15.0	17.7	15.3

**Table 2:** Parameters of the 3D unit cell.

Group	Samples	*L* (mm)	*R*_1_ (mm)	*R*_2_ (mm)	*ρ′*
1	11 (BCC)	4.0	0.35	0.35	0.140
	12	4.0	0.33	0.43	0.140
	13	4.0	0.30	0.53	0.139
	14	4.0	0.27	0.65	0.142
2	21 (BCC)	4.0	0.40	0.40	0.178
	22	4.0	0.375	0.50	0.177
	23	4.0	0.35	0.60	0.177
	24	4.0	0.325	0.70	0.179
3	31 (BCC)	4.0	0.30	0.30	0.106
	32	4.0	0.275	0.385	0.105
	33	4.0	0.25	0.475	0.106
	34	4.0	0.225	0.55	0.106

**Table 3:** J-C constitutive model and J-C fracture model parameters.

*d*_1_	*d*_2_	*d*_3_	*d*_4_	*d*_5_	Melting Temperature	Transition Temperature	*A*	*B*	*n*	*m*	*C*
−0.09	0.25	−0.5	0.014	3.87	1630	995	862	331	0.34	0.8	0.012

**Table 4:** Fabrication parameters.

Power (W)	Spot Diameter (mm)	Scan Space (mm)	Layer Thickness (μm)	Density (%)
200	0.1	0.15	50	99

**Table 5:** Relative density of fabricated samples.

Group	Samples	Sample Mass (g)	Relative Density
1	11 (BCC)	10.8	0.149
	12	11.0	0.152
	13	11.0	0.152
	14	11.3	0.156
2	21 (BCC)	13.6	0.187
	22	14.0	0.193
	23	13.8	0.190
	24	14.1	0.194
3	31 (BCC)	8.3	0.114
	32	8.6	0.118
	33	8.8	0.121
	34	8.8	0.121

**Table 6:** Relationship between *ρ*′, *ϕ*_1_ and initial stiffness, plastic failure strength of samples.

Group	Samples	*ϕ*_1_ (°)	*ρ*’	*σ*’ (MPa)	*E* (MPa)
1	11 (BCC)	106.65	0.140	30.3	385.4
	12	91.62	0.140	28.3	356.4
	13	79.94	0.139	26.1	325.2
	14	70.53	0.142	21.3	271.5
2	21 (BCC)	105.41	0.178	45.6	570.5
	22	93.17	0.177	43.9	547.0
	23	81.44	0.177	40.3	476.3
	24	70.53	0.179	34.0	412.8
3	31 (BCC)	102.8	0.106	17.4	215.4
	32	93.72	0.105	16.9	208.2
	33	81.38	0.106	16.1	185.6
	34	70.53	0.106	12.3	145.5
